# Vitamin D in Lupus Patients of Childbearing Age: Are We Doing Enough?

**DOI:** 10.3389/frph.2022.936810

**Published:** 2022-06-27

**Authors:** Gianina Statache, Sadaf Brown

**Affiliations:** Department of Rheumatology, Department of Family Medicine, Abu Dhabi Stem Cell Centre, Abu Dhabi, United Arab Emirates

**Keywords:** lupus, pregnancy, vitamin D, fertility, supplements, outcome

## Abstract

Systemic lupus erythematosus patients have long been observed to suffer from vitamin D deficiency. This can be related to either environmental factors, medication, or other comorbidities like renal disease. Moreover, lupus patients have reported conception issues including ovarian failure or recurrent miscarriages. There are vast data regarding vitamin D's ability to support the development of a healthy pregnancy and prevent complications, such as pre-eclampsia and gestational diabetes, likely through its ability to regulate both innate and adaptive immune systems. Although there is an agreement in the medical world that pregnant lupus patients should be screened and receive vitamin D supplements, there are no official guidelines on screening and often the recommended doses are suboptimal. Further research is needed to look at the potential of vitamin supplementation in pregnant lupus patients.

## Introduction

Systemic lupus erythematosus (SLE) is a chronic multisystem autoimmune condition mainly affecting women, with a 6–10-fold female predominance and mostly of childbearing age (1). Lupus pathogenesis is complex and still unknown. It includes an altered self-tolerance with innate and adaptive immune responses against self-antigens. Malfunction in the signaling, proliferation and activation of both B and T cells has been reported. Lupus patients have an increased expression of IFN gamma gene transcripts and poorly functional T regulatory cells (2).

Systemic lupus erythematosus has a broad spectrum of mild to severe symptoms with irreversible organ damage or death. Most common symptoms include arthralgia, skin rashes, cytopenia, serositis and kidney involvement. Less frequent vasculitis, cardiac, neurologic and eye involvement have been reported. Most of these symptoms respond to immunosuppressive treatment but can have a debilitating impact on the patients' quality of life.

Apart from the clinical picture, the lupus diagnosis is supported by the presence of autoantibodies. Although ANA antibodies can be found in 5%−10% of the general population, in SLE, anti-Ro, anti-La, and anti-Sm antibodies have higher specificity and can be found in various percentages between 20 and 40% (3, 4). Anti-Ro antibodies are particularly of interest as they are associated with congenital heart block and neonatal lupus, whilst dsDNA titres correlate with disease activity. Other antibodies detected in SLE patients are antiphospholipid antibodies found in 40% of patients and associated with thrombotic events and pregnancy morbidity (5).

Various factors have been named to contribute to lupus pathogenesis, including genetic, hormonal, or environmental factors.

This review aims to present recent research progress on the benefits of vitamin D supplementation in lupus patients that are pregnant or planning pregnancy.

## Lupus and Pregnancy

Hormonal factors have been mainly studied due to the high prevalence of cases in young women. This was further supported by several studies showing an increase in flares in patients using contraceptive measures (6). However, Petri et al. have demonstrated in a group of 183 women using a combined estrogen birth-control during the 12 months follow up period; that there was no increase in the number of flares. It was suggested that this type of contraception should be considered for women needing birth-control measures (7).

Despite recent advances in treatment options and over improvement in the rate of complications and death, lupus remains a condition with higher morbidity than the general population (8). In the US, lupus remains one of the leading causes of death in young women. Maternal death can be 20 times higher in lupus patients due to various complications, including infection, thromboembolic events, and pulmonary hypertension (9, 10).

In the 80s, in lupus patients, pregnancies were contraindicated due to the high risk of complications for the mother and baby (2). Since then, the progress made in understanding the disease and improving treatment has led to more women planning pregnancy. It is essential to counsel patients about reasonable disease control before pregnancy, as flares arise in 30%−60% of pregnant patients. It is more common in those with active disease at the beginning of the pregnancy (11). Given the potential risk for complications, there are a few absolute contraindications to pregnancy like pulmonary hypertension, severe renal involvement, and severe heart failure. The presence of any of these complications should lead to discussions with the patient and family regarding the high risk to both the mother and the baby.

It has been long cited that lupus patients have difficulties conceiving due to delays in starting a family until the disease is better controlled or until after the medication washout period. Lupus patients also suffer from menstrual disturbances associated with high inflammation levels linked to high follicle-stimulating hormone levels (12). Moreover, the medication used in lupus management has also been linked with infertility, for example, cyclophosphamide which increases the risk of ovarian failure or steroids that have been associated with menstrual disturbances (13).

Out of all identified auto-antibodies, antiphospholipid antibodies are often the cause of repeated pregnancy losses, intrauterine death, and preterm delivery. Antiphospholipid syndrome (APS) or Hughes syndrome is defined by anti-cardiolipin antibodies, beta-1 glycoprotein, and lupus anticoagulant on two separate occasions 12 weeks apart (14). APS has been found in up to 40% of women with repeated pregnancy loss, and it can complicate lupus in about 40% of cases. Triple positive cases are more likely to suffer complications, and they have a lower chance of live birth (30%) (15).

Anti-Ro antibodies can actively pass the placenta and cause heart block or neonatal lupus syndrome. Monthly scans are recommended during pregnancy to detect the heart block or progression to cardiac failure. There is no known intervention to reverse the heart block. However, the use of hydroxychloroquine during pregnancy has been shown to reduce the risk (16) significantly.

Recognition and management of SLE flares during pregnancy can be particularly challenging as some features can change. For example, complement levels and erythrocyte sedimentation rate can rise, whilst a certain degree of anemia and proteinuria are considered physiological during pregnancy (17). The severity of the symptoms guides SLE treatment during pregnancy. However, many drugs commonly used are contraindicated during pregnancy due to the increased risk of fetal malformations. The European League Against Rheumatism and the British Rheumatology Society have developed a set of recommendations regarding the use of these drugs before and during pregnancy (18).

## Vitamin D and Pregnancy

Classically vitamin D's role is in regulating the balance between calcium and phosphorus. However, vitamin D is implicated in other processes like regulation of cell proliferation, inflammatory response, and genome stability. Vitamin D insufficiency has been correlated with poor pregnancy outcomes, including pre-eclampsia, miscarriages or gestational diabetes.

The vitamin D receptor (VDR) is expressed in the placenta, decidua, and endometrial stromal cells during pregnancy, suggesting an essential role in immunomodulation and possibly embryo implantation (19). The innate immune system is stimulated to prevent rejection of the fetal allograft, whilst the adaptive immune response is suppressed. The maintenance of maternal-fetal tolerance is achieved through the involvement of macrophages and T cells. Vitamin D has a role in converting M1 macrophages into M2 macrophages. Whist M1 macrophages dominate the initial phases of embryo implantation, an M1/M2 balance in the favor of M2 is essential for normal fetal development (20).

Some research has suggested that vitamin D can prevent pre-eclampsia after noticing that vitamin D metabolism is altered in the placenta of pre-eclamptic patients (21). Several studies demonstrate that women with severe preeclampsia had significantly lower vitamin D levels than normotensive women (90% vs. 62%). Moreover, pre-eclamptic women had a higher incidence of low birth weight and higher chances of undergoing a cesarean section when vitamin D levels were below 15 ng/ml (22). It is still unclear how vitamin D is involved in the development of preeclampsia, but it has been suggested that low vitamin D can cause epithelial cell dysfunction (23).

Insulin resistance changes throughout pregnancy, with the highest levels occurring in the last trimester. Vitamin D contributes to insulin sensitivity and is involved in insulin production (24, 25). A Chinese study has shown that the risk of gestational diabetes is 1.8-fold higher in women with vitamin D deficiency. Another study demonstrated that low vitamin D levels were associated with insulin resistance (26, 27).

## Vitamin D and Immune Regulation

**Figure 1:**
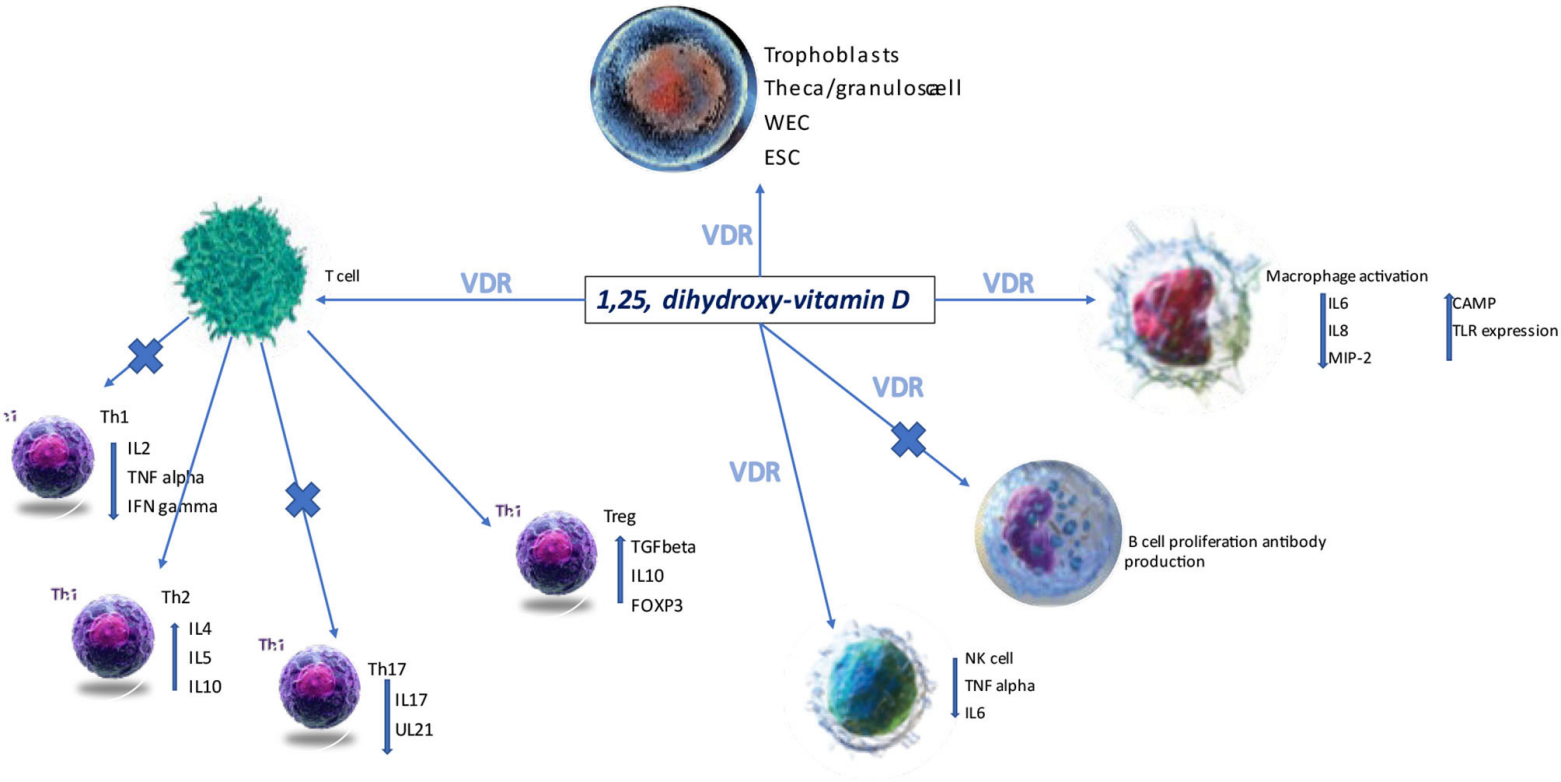
VDR, vitamin D receptor; ESC, endometrial stem cells; NK, natural killer cells; Th, T helper; WEC, whole endometrium cells (28).

The broad expression of the vitamin D receptor (VDRE) on various cell lines suggests its ability to regulate the immune system (Figure 1). In pregnancy and particularly during the early phases of embryo implantation, prevention of a pro-inflammatory state is essential in achieving maternal-fetal tolerance.

1,25 hydroxycholecalciferol polarizes the immune response to a Th2 response, and it is vital to the expression of the transcription factor forkhead box P3 (FOXP3) via VDRE (29). Moreover, vitamin D has been reported to down or upregulate various cytokines (IL-6, IL-10, IL-2) secreted by dendritic cells, T cells, T regulatory cells or macrophages (30). Its role in controlling the innate immune system has been suggested to explain recurrent miscarriages, likely due to an increase in the number of CD19 B cells and CD56 NK cells and the titres of circulating autoantibodies. Vitamin D deficiency is more frequent in these groups of patients (13% had levels <20 ng/ml). Vitamin D supplementation can correct this dysregulation, and this was supported by finding in human studies where the percentage of CD19 B cells and TNFα T cells were both reduced (31, 32).

### Vitamin D and Lupus Activity

Vitamin D deficiency is notorious in lupus patients. It is still debatable if low vitamin D is a consequence of the inflammatory state or is responsible for the high disease activity (33). Unfortunately, the uncontrolled disease can lead to kidney damage, characterized by heavy proteinuria.

Vitamin D usually binds with DBP (vitamin D binding protein) 85%−90% or albumin 10%−15%. A very small amount (less <0.03%) exists in free from call free vitamin D (34). In lupus patients with kidney involvement, there is a loss of DPB which can contribute to low vitamin D levels due to excess loss in the urine (35). Free vitamin D levels are not influenced by liver or kidney function or hormonal levels, hence the measurement of free vitamin D in this category of patients might be a better marker to assess vitamin D bioavailability and can potentially offer an explanation regarding the controversial data reported so far regarding low levels of vitamin D and disease activity.

A study by Lermar et al. (36) showed that the addition of vitamin D to cell cultures incubated with sera from SLE patients had reduced the expression of CD40 and CD86 and increased the expression of CD14. Research studying the cytokine pattern in SLE patients has pointed to overexpression of type I interferon (37). However, a randomized control trial (RCT) run by Aranow et al. (38) failed to suppress the interferon signature. Other studies have proven that vitamin D supplementation reduces disease activity, translating into fewer Th1 and Th17 cells and lower titres of dsDNA (33, 39).

Petri et al. (40) showed that 1,25hydroxycholecalciferol has minimal impact on disease activity and urinary protein excretion. The Systemic Lupus International Collaborating Clinics (SLICC) group showed a connection between low vitamin D and hypertension; a metanalysis of extensive studies failed to prove a connection (41, 42).

There is a greater need for randomized control trials looking at the possible benefits of supplementing vitamin D in lupus patients. Most trials to date are small, and results do not always consider confounders like concomitant medication, lifestyle, and other comorbidities. Moreover, there is no agreement on the appropriate dose to use to achieve disease control. Interestingly most studies on animal models that reported a connection had used 25(OH)D3, which is the active form, as opposed to cholecalciferol (inactive form), most commonly prescribed in practice (43, 44).

The relevant studies looking at the impact of vitamin D on lupus disease activity are presented in Table 1.

**Table 1:** Studies looking at vitamin D treatment and disease activity.

**Study**	**Type of study**	***n***	**Vitamin D dose**	**Outcomes**	**Reference**
Magro et al.	Observational	31	8,000 IU daily for vitamin D deficiency; 8,000 IU daily for 4 weeks for vitamin D insufficiency, followed by 2,000 IU daily as a maintenance dose	improved disease activity as measured by SLEDAI-2K, *p* = 0.028; Improved fatigue measured by fatigue severity scale (FSS; *p* = 0.071)	(45)
Lima et al.	RCT, 24 weeks	40	50,000 IU weekly	significant improvement in SLEDAI (*p* = 0.010), ECLAM (*p* = 0.006)	(46)
Terrier et al.	Cross-sectional, 6 months	24	100,000 IU weekly for 4 weeks followed by 100,000 IU monthly for 6 months	Increase in Treg absolute numbers at month 2 (*p* < 0.001), decrease in Th1 and Th17 at month 6 (*p* < 0.05, *p* < 0.81); decrease in SLDAI at month 6 (*p* = 0.16)	(39)
Petri et al.	observational study	1,006	50,000 IU weekly plus 200IU vitamin D and calcium daily	decrease in SLEDAI (*p* + 0.65), decrease in proteinuria (*p* < 0.001), decrease in dsDNA titres (*p* = 0.78)	(40)
Ruis-Irastorza et al.	Cross-sectional, 6 months	60	600–800 IU daily	improvement in fatigue (*p* + 0.015), decrease in SLEDAI (*p* = 0.015)	(47)
Aranow et al.	RCT	57	2,000 IU, 4,000 IU and placebo	No change in IFN signature in inactive disease, safety	(38)

*RCT, randomized controlled trial; IU, international units; IFNγ, interferon-gamma; SLEDAI, systemic lupus erythematosus disease activity index; ECLAM, European Consensus Lupus Activity Measurement*.

### Vitamin D Supplementation in Pregnant Lupus Patients

Currently, screening for vitamin D deficiency is not recommended in pregnant women with or without SLE.

There is a standard agreement that we should offer 25(OH)D to all pregnant women with lupus; however, in clinical practice, we fail to screen these patients, and this is particularly important in those with long-term use of steroids, where data revealed that vitamin D acts as a steroid-sparing agent (48–50). Another group of patients that should be prioritized in receiving supplements are those with APS. *In vitro*, vitamin D inhibits the tissue factor and adhesion molecules in endothelial cells exposed to beta-2-glycoprotein antibodies (51). Moreover, low vitamin D levels have been correlated with higher thrombosis events (52).

Pregnant lupus patients with secondary APS, just like those with primary APS, are being treated prophylactically with low molecular weight heparin (LMWH) alone or in combination with aspirin (53). However, clinical studies have shown contradictory evidence on the benefits of this regimen in modifying pregnancy outcomes, including some detrimental effects on placental neo angiogenesis by increasing the circulating levels of fms-like tyrosine kinase 1 (sFlt-1) (53, 54). Interestingly vitamin D appears to inhibit the release of sFlt-1. Hence, vitamin D supplementation in pregnant patients receiving LMWH could counteract the potential detrimental effects on the placenta.

Positive ANA antibodies have long been linked with recurrent pregnancy loss (RPL). In a recently published metanalysis, it was found that up to 20% of RPLs are found to have positive ANA antibodies. Higher titres and a homogeneous pattern had a stronger association, whilst detected dsDNA presence did not show a significant correlation (55). It is believed that ANA increases the risk of miscarriage by activating the immune system and inducing placental insufficiency (56).

Vitamin D deficiency was found in 45% of RPLs cases, and these patients have a higher incidence of a positive ANA test (31).

More than 70% of lupus patients have a positive ANA on screening. These autoantibodies are related to the abnormalities in T and B cell functions, including Th1, Th2 and Treg dysregulations. In pregnant women with vitamin D deficiency, Treg activity is disrupted. Vitamin D supplementation has increased Treg number, Th17 cell number, and IL10 production (57).

There is no agreement on the right dose necessary to correct the deficiency in this category of patients. Although numerous papers support the safety of higher doses of vitamin D, in standard practice doses are often suboptimal and do not exceed 2,000 IU per day (58–60). In lupus patients, higher doses associated with regular monitoring have been found to improve disease activity and were generally well tolerated (45).

## Discussion

The evidence for immune involvement in lupus is well documented, and research increasingly confirms that immune regulation is impacted in vitamin D deficiency in favor of an increased inflammatory environment. Similarly, vitamin D supplementation has been linked with reduced inflammation in the immune system. In pregnancy, vitamin D deficiency has been associated with increased complications such as pre-eclampsia and miscarriage, with supplementation associated with the inverse trend.

Lupus patients with active disease at conception are already predisposed to such complications, including, miscarriage, RPL and thrombosis- especially in APS. In these patients, vitamin D deficiency has been found to correlate with positive lupus antibodies and RPL, with supplements shown to reverse this in some studies. However, findings from other research show the impact of vitamin D in lupus patients is not significant and the relationship with complications including elevated blood pressure and pre-eclampsia is less clear.

In reality, the relationship between vitamin D deficiency, lupus disease and pregnancy is likely to be complex. Current conclusions are based on a low number of clinical studies, mostly underpowered and with poor trial design, which may be further impacted by variables such as ethnicity, body mass index, diet, and concomitant medication, in addition to comorbidities such as renal disease. It is also unclear from this research whether vitamin D deficiency perpetuates lupus activity or if the disease itself prevents adequate vitamin D production or absorption. Further research is needed to clarify these interactions and subsequently guide interventions, including supplementation and lifestyle modifications during pregnancy.

The impact of pregnancy and lupus combined on therapeutic levels of vitamin D and therefore the question of adequate dosage also remain unanswered and in need of further investigation.

A recommended change in clinical practice could include regular screening for vitamin D deficiency in patients with lupus, with appropriate supplementation and observation of the impact of pregnancy on levels and therapeutic requirements. Measuring free levels as opposed to total levels of vitamin D might also give some insight into true levels of deficiency, though this is likely to be a costly intervention.

Ultimately, higher-powered studies are needed to qualify the outcome of vitamin D supplementation on pregnancy in a patient with lupus, with further pre-clinical research required to explore the exact immunological effects.

## Author Contributions

GS and SB both contributed equally in writing and reviewing the paper. All authors contributed to the article and approved the submitted version.

## Conflict of Interest

The authors declare that the research was conducted in the absence of any commercial or financial relationships that could be construed as a potential conflict of interest.

## Publisher's Note

All claims expressed in this article are solely those of the authors and do not necessarily represent those of their affiliated organizations, or those of the publisher, the editors and the reviewers. Any product that may be evaluated in this article, or claim that may be made by its manufacturer, is not guaranteed or endorsed by the publisher.
